# Simple and sensitive colorimetric sensors for the selective detection of Cu(ii)†

**DOI:** 10.1039/d0ra09910d

**Published:** 2021-03-23

**Authors:** Meifang Liu, Kequan Wang, Hanlu Wang, Jie Lu, Shukang Xu, Lulu Zhao, Xilong Wang, Junming Du

**Affiliations:** College of Chemistry, Chemical & Environmental Engineering, Weifang University Weifang 261061 P. R. China liumf@iccas.ac.cn; Weifang Environmental Monitoring Center China; Guangdong Provincial Key Laboratory of Petrochemical Pollution Process and Control, Guangdong University of Petrochemical Technology China; Harway Pharma Tech Co., Ltd. China

## Abstract

A simple, sensitive colorimetric probe for detecting Cu(ii) ions with fast response has been established with a detection limit of 2.82 μM. UV-Vis spectroscopy along with metal ion response, selectivity, stoichiometry, competition was investigated. In the presence of copper(ii), the UV-Vis spectrum data showed significant changes and the colorimetric detection showed a color change from colorless to yellow. After the selective binding of receptor L with Cu(ii), the UV-visible absorption at 355 nm decreased dramatically, a new absorbance band appeared at 398 nm and its intensity enhanced with the increase in the amount of Cu(ii). Moreover, it exhibited highly selective and sensitive recognition towards Cu(ii) ions in the presence of other cations over the pH range of 7–11. The complex structure was verified by FT-IR spectroscopy, elemental analysis and quantum mechanical calculations using B3LYP/6-31G(d) to illustrate the complex formation between L and Cu(ii). According to the Job plot and the quantum mechanical calculations, the stoichiometric ratio for the complex formation was proposed to be 1 : 1.

## Abstract 2

Cu(ii) ion with L led to a rapid and sensitive color change from colorless to yellow.

## Graphical abstract

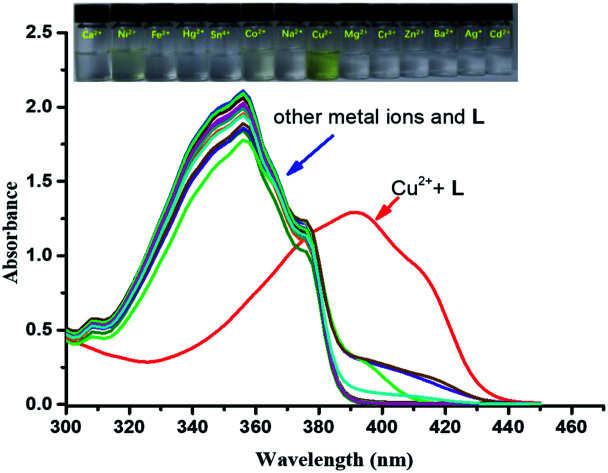

## Introduction

Over the past decades, there has been considerable interest in the development of chemosensors for detecting heavy metal ions as they are harmful to the environment and important biological processes.^1–7^ As the third abundant element, copper(ii) ion plays vital roles in numerous physiological processes. However, either excess or deficient amounts may aggravate the deterioration of vital organs and lead to the progression of complications. When humans and animals are over-exposed to Cu(ii)-contaminated water, copper ions tend to accumulate, which is toxic to the environment and ecosystem, and also has an impact on the progress of Alzheimer's disease and Parkinson's disease.^8–10^ Therefore, numerous methods have been developed for the trace detection of Cu(ii) in biological and environmental specimens, such as inductively coupled plasma mass spectrometry (ICP-MS),^11,12^ atomic absorption spectrometry (AAS),^13,14^ voltammetry,^15,16^ and total reflection X-ray fluorimetry (TXRF).^17–20^ Among the common analytical methods, most of them often require expensive instruments and sophisticated detection systems. At present, fluorescence spectroscopy is a frequently used technique for the detection of copper(ii) ions in the biological and environmental media. In many cases, fluorescent sensors have limitations due to fluorescence quenching of numerous metal ions.^21–26^ Cu(ii) also exhibits fluorescence quenching due to its intrinsic paramagnetic nature.^27–29^ In practical applications, it is necessary to develop a simple, convenient, and low-cost Cu(ii) sensor. Therefore, the design and synthesis of colorimetric sensors for the detection of Cu(ii) is very valuable.

Chemosensors for the selective detection of Cu(ii) generally have structures of chelating metal ions with heteroatoms (nitrogen and sulfur), such as naphthalimide,^30–32^ naphthalene,^33–35^ anthracene,^36^ anthraquinone,^37^ quinoline,^38–41^ thiazole,^42^ and macrocyclic^43–45^ derivatives. However, many of them are obtained *via* complex synthesis procedures or require expensive materials. For example, Zhang *et al.*^46^ reported that the synthesis of a naphthalimide derivative required six steps. Therefore, developing novel chemosensors for detecting metal ions with synthesis and commercial availability are necessary. In this study, chemosensor L based on a pyridyl-isoindoline-1-one skeleton was easily obtained with good yield using simple and inexpensive starting materials, which manifested highly sensitive and selective colorimetric detection towards Cu(ii) with a color change from colorless to yellow in a mixed solvent. The complex skeleton was verified by FT-IR spectroscopy, MALDI-TOF, elemental analysis and quantum mechanical calculations to illuminate the formation of the complex between Cu(ii) and L; the stoichiometric ratio of 1 : 1 for complex formation and mechanism for the detection of the Cu^2+^ ion with L were proposed.

## Experimental

All reagents were of analytical grade and used without purification. The inorganic salts of Fe^2+^, Ag^+^, Mg^2+^, Na^+^, Co^2+^, Ca^2+^, Cr^3+^, Cd^2+^, Hg^2+^, Ni^2+^, Ba^2+^, Zn^2+^, Cd^2+^, and Cu^2+^ ions and 2-pyridylaldehyde and isoindoline-1,3-dione were purchased from Chemical Reagent Company.

Absorption spectra were recorded on a TU-1901 double beam UV-Vis spectrophotometer. While ^1^H NMR spectra were obtained on a Bruker Avance 600 MHz NMR. CD_3_OD and CDCl_3_ were used as the solvent. FT-IR and elemental analyses were conducted on a Bruker Alpha spectrometer and Elementar Vario EL CUBE, respectively. MALDI-TOF was performed on a Bruker Autoflex speed TOF/TOF mass spectrometer using dithranol (cas: 1143-38-0) as the matrix. Quantum chemistry calculations at the density functional theory of B3LYP were used to fully optimize two molecules, and the calculations were completed in Gaussian09.

### Elemental analysis

Anal. calcd for C_14_H_9_CuN_3_O_4_: C, 48.49; H, 2.62; N, 12.12; found: C, 48.43; H, 2.29; N, 12.05.

## Results and discussion

**Scheme 1:**
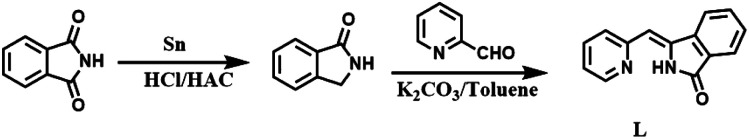
Synthesis of compound L.

Receptor L was synthesized according to the literature method.^47^ The synthetic routes of compound L are displayed in Scheme 1. Starting from isoindoline-1,3-dione, the immediate precursor isoindolin-1-one was synthesized with a 80% yield. Upon heating a 1 : 2 : 4 mixture of isoindolin-1-one, 2-pyridylaldehyde and K_2_CO_3_ at 110 °C for 24 h in toluene, compound L was obtained in 85% yield and characterized *via* FT-IR and ^1^H NMR spectroscopy techniques.

**Fig. 1:**
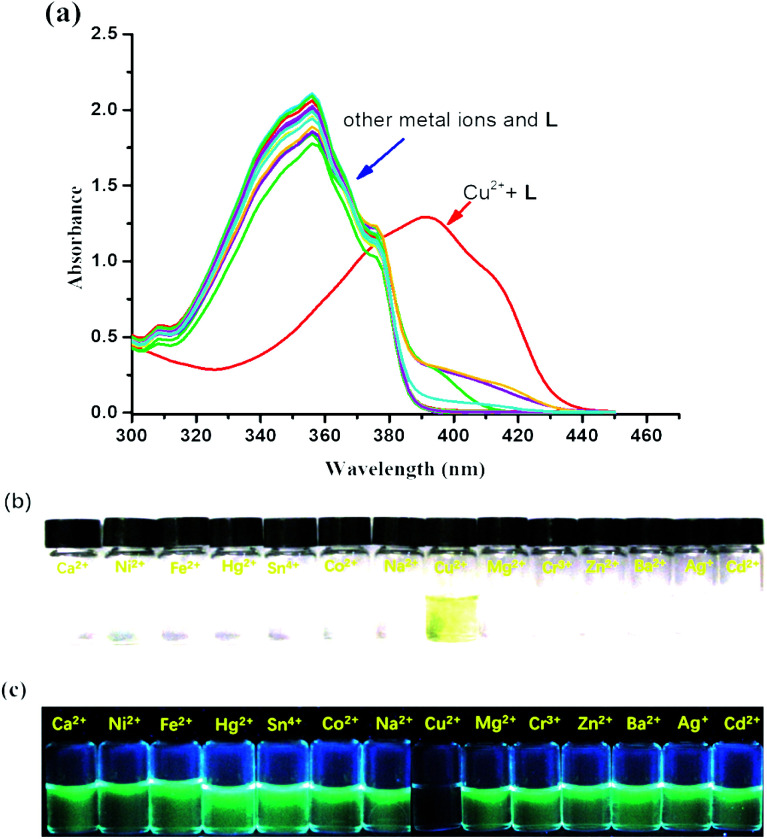
(a) UV-Vis spectral changes of receptor L (50 μM) after the addition of 2 equiv. of Cu^2+^ and 5 equiv. of other metal ions in the EtOH/H_2_O solution (v/v, 4 : 1). (b) The color changes of L (50 mM) upon the addition of various metal ions (5 equiv.) in the EtOH/H_2_O solution (v/v, 4 : 1). (c) Fluorescent changes of L (50 μM) upon the addition of various metal ions (5 equiv.) in the EtOH/H_2_O solution (v/v, 4 : 1).

The colorimetric selective sensing ability of receptor L with various cations in the EtOH/H_2_O (v/v, 4 : 1) mixed solution was determined by UV-Vis absorption spectroscopy (Fig. 1a). It was found that the absorption maximum of L was located at 355 nm in the absence of metal ions. Only after the addition of 2.0 equiv. of Cu^2+^, the absorption maximum of the complex L–Cu(ii) red-shifted to 398 nm; however, other cations such as Co^2+^, Mg^2+^, Na^+^, Ca^2+^, Cr^3+^, Ba^2+^, Fe^2+^, Zn^2+^, Co^2+^, Hg^2+^, Ni^2+^, Cd^2+^, and Ag^+^ combined with L had no influence on the spectra. The new absorption band was attributed to the metal-induced intramolecular charge transfer from receptor L to the Cu^2+^ ion.^48,49^ Compared with the photograph of receptor L with various cations (Fig. 1b), the solution of L with Cu^2+^ ions caused a rapid and sensitive color change from colorless to yellow, which indicated that the receptor L could be used as a ‘naked-eye’ indicator for copper ions in mixed solutions. In addition, chemosensor L for Cu^2+^ can strongly quench the fluorescence of a fluorophore by the PET mechanism^50^ (Fig. 1c).

**Fig. 2:**
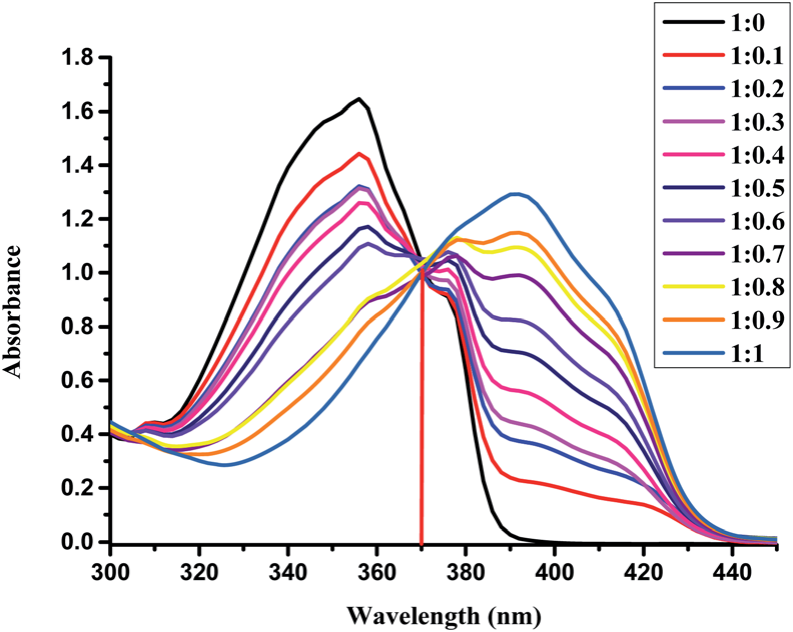
UV-Vis spectral changes of L (50 μM) upon the addition of Cu^2+^ (from 0 up to 1 equiv.) in the EtOH/H_2_O (4 : 1) solution at room temperature.

The detection limit of L for the analysis of Cu(ii) was investigated by UV-Vis absorption spectra of L (5.0 × 10^−5^ M) in an EtOH/H_2_O = 4 : 1 solution upon addition Cu^2+^ at room temperature, as shown in Fig. 2 (Fig. S1 in ESI†). With the addition of Cu^2+^, the absorption band at 355 nm decreased gradually, while the absorption band at 398 nm increased and reached its maximum at 1 equiv. of Cu^2+^. The isosbestic points at 370 nm indicated that a single species between the Cu^2+^ and receptor L was formed, and the maximum absorption peak of the spectrum could be redshifted by 43 nm, and the color changed from colorless to yellow. At 398 nm, the band gap with a molar extinction coefficient of 1.2 × 10^4^ M^−1^ cm^−1^ (±10%), is not Cu-based d–d transitions due to the ligand-to-metal charge-transfer (LMCT) mechanism.^51^ By calculation, the detection limit of L for the analysis of Cu(ii) was 2.82 μM with the linear working range from 20 μM to 45 μM, which was determined from the plot of the relative absorbance intensity *I*_398 nm_/*I*_355 nm_ as a function of the concentration of Cu(ii) on the basis of 3*σ*/*k* according to the literature^52,53^ (Fig. S2 in ESI†). Compared to other recently reported Cu(ii)-sensors, the detection limit of our sensor was higher, as shown in Table S1.†^54–60^ Although some of the reported sensors provided better detection limits, some proceeded with the “turn-off” mode for the detection of Cu(ii) and had inferior selectivity compared to that of our sensor in this study.

**Fig. 3:**
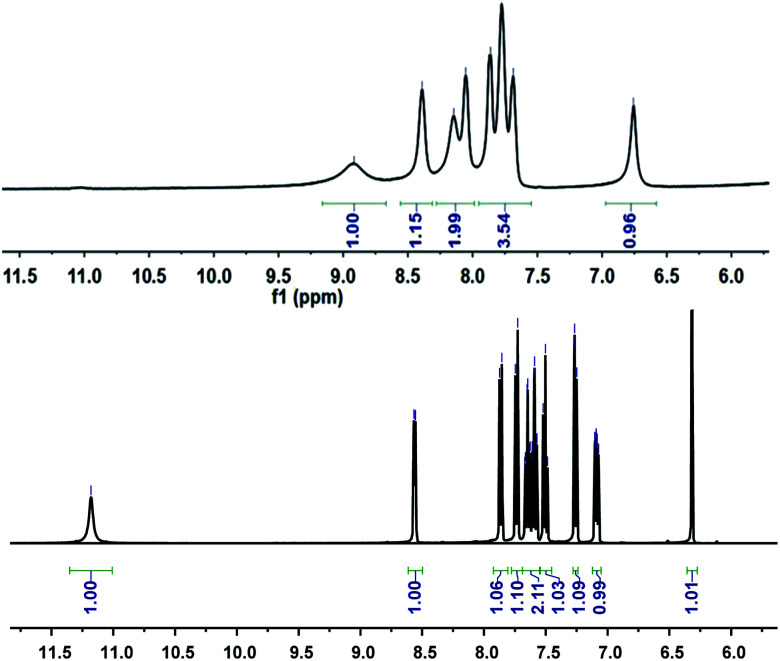
^1^H NMR spectrum of L and L with Cu^2+^ (ratio 1 : 1).

**Fig. 4:**
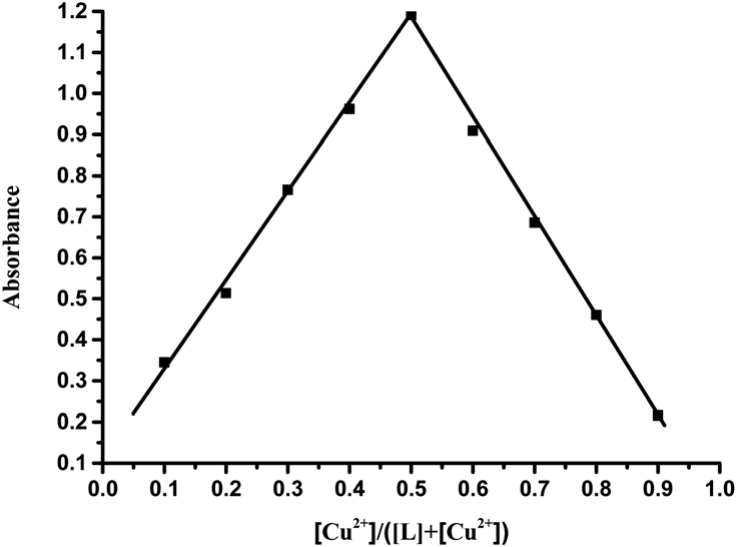
Job plot for the binding of L with Cu^2+^. Absorbance at 398 nm was plotted as a function of the molar ratio [Cu^2+^]/([L] + [Cu^2+^]). The total concentration of Cu^2+^ with receptor L was 5.0 × 10^−5^ M.

**Fig. 5:**
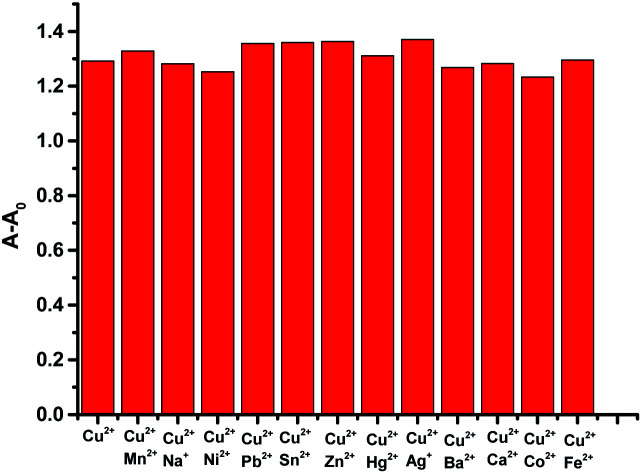
UV-Vis selectivity–competition study of receptor L with added M(ii) salts (5.0 equiv.), followed by the addition of 1.0 equiv. of Cu(ii). [L] = 5.0 × 10^−5^ M, absorbance changes monitored at 398 nm. Relative intensity of L (0.01 mM) in presence of various metal ions in the EtOH/H_2_O solution, detection wavelength: 398 nm.

Further study of the features of L–Cu(ii) interactions was obtained by ^1^H NMR experiments in the CD_3_OD solvent (Fig. 3). By hydrogen spectrum analysis, the chemical shift of the complex L–Cu(ii) changed greatly after the addition of copper ions. Compared with ^1^H NMR before and after the addition of copper ions, the N–H chemical shift at 11.18 ppm became weak, and the signal of C–H also slightly became broader. It is suggested that nitrogen in secondary amides might be involved in the binding of Cu^2+^, which was consistent with the previous reports.^61,62^ The stoichiometry of L was obtained by the Job's plot. The results showed that when the molar ratio was 0.5, and the absorbance band of L–Cu(ii) complex reached the maximum, which indicated that the stoichiometric ratio of the complexes between L to Cu^2+^ ions was 1 : 1 (Fig. 4 and S3 in ESI†). In order to research the effects of other metal ions on the complex of Cu(ii)–L, competitive experiments were conducted by the addition of copper ions (1 equiv.) to the solution of L with other metal ions (5 equiv. of Na^+^, Co^2+^, Mg^2+^, Ca^2+^, Ba^2+^, Cr^3+^, Fe^2+^, Co^2+^, Hg^2+^, Ni^2+^, Zn^2+^, Cd^2+^, and Ag^+^). As shown in Fig. 5 (Fig. S4 in ESI†), compared with the addition of Cu(ii) to the solution of L, the presence of competing metal ions did not cause any distinct change in UV-Vis absorption spectra. Thus, this displayed the stronger binding capacity of L towards Cu(ii), and simple receptor L could be used as a preferential selective colorimetric sensor for Cu(ii) in the presence of most interfering ions.

**Fig. 6:**
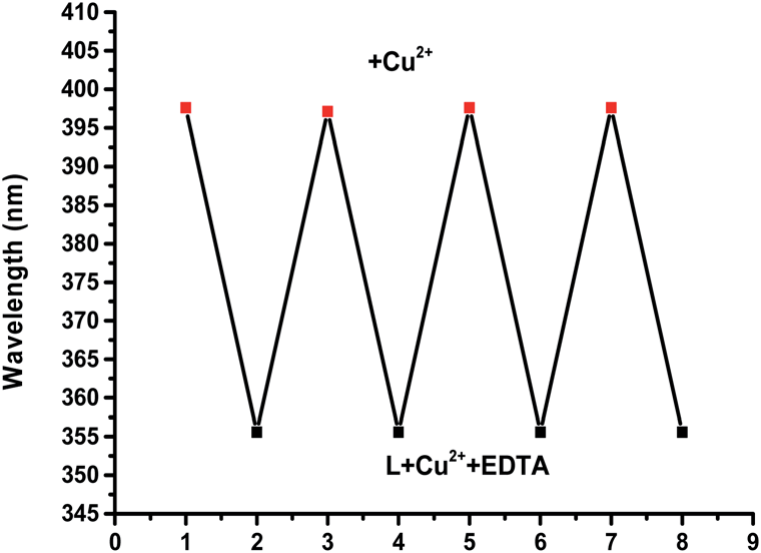
The cycle of L–Cu^2+^ and L + Cu^2+^ + EDTA.

We next investigated the effects of pH on the response of complex L towards Cu(ii) ions. It was clear that the absorption spectrum of the ligand was almost unchanged and the proposed L–Cu(ii) sensor could be applied successfully over the pH range 7–11 (Fig. S5 in ESI†). With pH greater than 11, the precipitation of Cu(OH)_2_ likely occurred in these conditions.^63,64^ With pH less than 7, due to the protonation process, the absorption peak of the complex L–Cu^2+^ at 398 nm decreases accordingly. In addition, to determine the repeatability and reproducibility of the L–Cu(ii) complex, a reversibility experiment was carried out with the addition of EDTA, which had a stronger binding ability towards Cu^2+^. It can be seen that with the addition of Cu^2+^ to L the maximum absorption band appeared at 398 nm, as shown in Fig. 6 (Fig. S6 in ESI†). After the addition of EDTA, the maximum absorption band of the complex appeared at 355 nm, and further addition of Cu^2+^ restored the location of the maximum absorption peak at 398 nm.

Quantum chemistry calculations at the optimized geometries of the free L and the L–Cu^2+^ complexes were conducted with the density functional theory of B3LYP to fully optimize the molecule, with a mix of basis sets composed of the split valence double-ζ (DZ) basis set 6-31G(d) for C, O, N, and H atoms, and effective core potential LANL2DZ for the Cu atom. DFT calculations indicated that the HOMO and LUMO of L were delocalized in the whole π-moiety and there was little difference between them (Fig. S7†). The band gap between HOMO (−5.7 eV) and LUMO (−2.0 eV) of L was calculated as 3.70 eV. On the other hand, the HOMO orbital electrons of L–Cu(ii) were uniformly distributed in the π-moiety of the molecule and the LUMO orbital electrons of L–Cu(ii) mainly delocalized around the nitrogen and oxygen atoms and copper ions. In addition, the band gap between HOMO (−2.98 eV) and LUMO (−2.33 eV) of the L–Cu(ii) complex was decreased to 0.65 eV. The smaller energy led to a large-scale spectral redshift, which met the requirement for colorimetric detection from colorless to yellow.

**Fig. 7:**
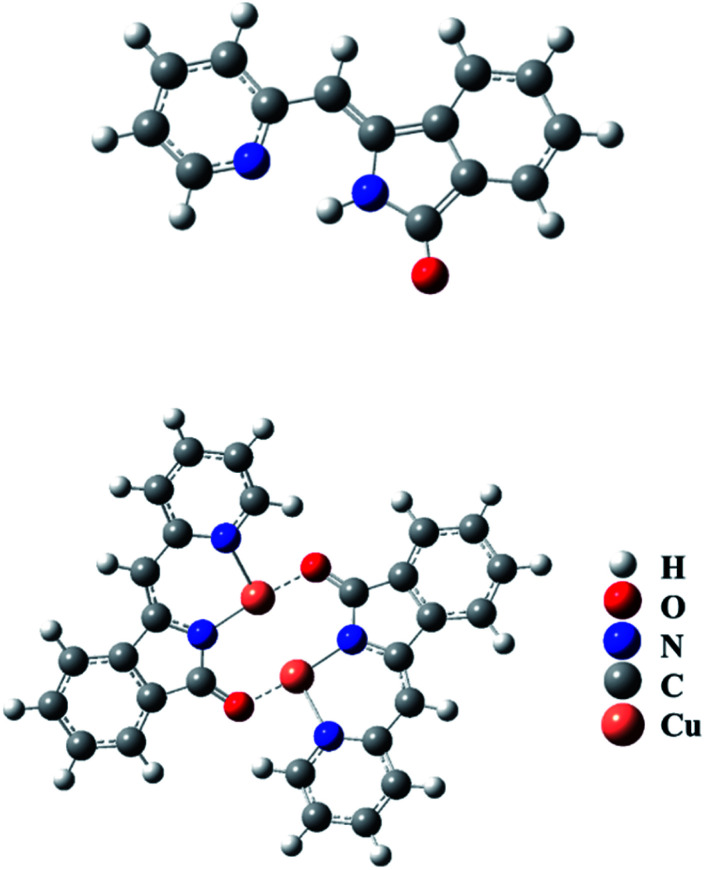
The structure of L and L–Cu^2+^ complexes optimized with density functional theory of B3LYP.

**Scheme 2:**
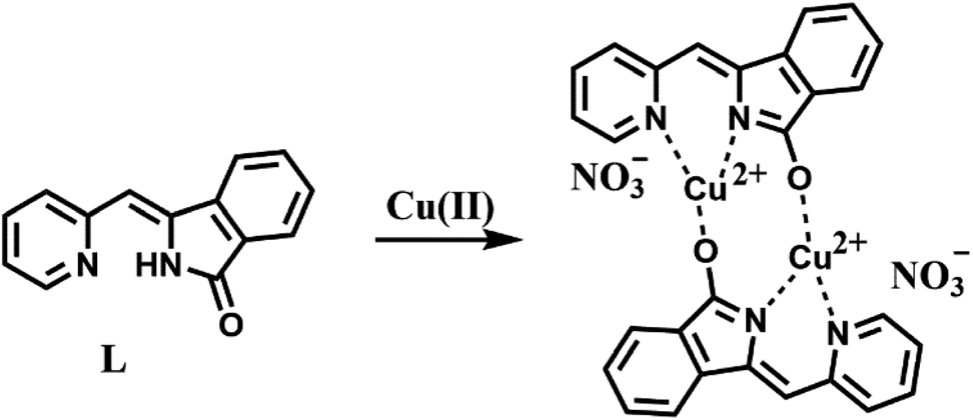
Proposed mode of complexation between L and Cu^2+^.

To explain a mechanism for the detection of Cu^2+^ ions with L, the FT-IR spectroscopy, MALDI-TOF, and elemental analysis were carried out. The FT-IR spectrum analysis showed the N–H stretching band at 3241 cm^−1^, and C=O stretching band at 1705 cm^−1^ in compound L. For compound L–Cu(ii), it presented the C=N stretching band at 1599 cm^−1^, and no N–H and C=O stretching bands. Elemental analysis showed that the molecular formula of the compound L–Cu(ii) is C_14_H_9_CuN_3_O_4_, and the molecular formula obtained was complex L–Cu(ii) combined with an anionic nitrate. From the analysis of MALDI-TOF, the cluster peaks at 568.838 and 567.829 corresponded to [L + Cu^2+^ + H^+^] (calcd = 569.008), [L + Cu^2+^] (calcd = 568.002), which indicated that the complex L–Cu(ii) has a bimolecular chelating center [L–Cu^2+^–L–Cu^2+^]. The binding mode of compound L with Cu^2+^ supported by the quantum chemistry calculation at the optimized geometries is shown in the Fig. 7. Based on the results from IR, elemental analysis, ^1^H NMR titration studies and quantum chemistry calculation, the proposed binding mode of ligand L with copper ions is through two nitrogen atoms and oxygen atom of carbonyl groups connected to adjacent molecules, as shown in Scheme 2.

## Conclusions

In conclusion, we provided a chemosensor sensor L with high yield by using simple starting materials, which manifested highly sensitive and selective colorimetric detection towards Cu(ii) with a color change from colorless to yellow. The experimental results displayed the stoichiometric ratio of 1 : 1 for complex formation were consistent with the quantum chemistry calculations.

## Conflicts of interest

There are no conflicts to declare.

## Supplementary Material

RA-011-D0RA09910D-s001
