# Supplementary material for: Simple and sensitive colorimetric sensors for the selective detection of Cu(ii)
Source: RSC Adv. 2021 Mar 23;11(19):11732–8. doi: 10.1039/d0ra09910d (PMC8695964; doi:10.1039/d0ra09910d)
Supplement: RA-011-D0RA09910D-s001 [file RA-011-D0RA09910D-s001.pdf]

Electronic Supplementary Material (ESI) for RSC Advances.
This journal is © The Royal Society of Chemistry 2021

Electronic Supplementary Information (ESI) for:

Simple and Sensitive Colorimetric Sensors for the Selective Detection of Cu(II)

Meifang Liu, [a]* Kequan Wang, [b] Hanlu Wang, [c] Jie Lu, [a] Shukang Xu, [a] Lulu Zhao, [a] Xilong Wang [d] and Junming Du [d]

[a] College of Chemistry-Chemical & Environmental Engineering, Weifang University, Weifang, 261061, P. R. China
E-mail: liumf@iccas.ac.cn (M. Liu)
Phone/Fax: +86-536-8785283

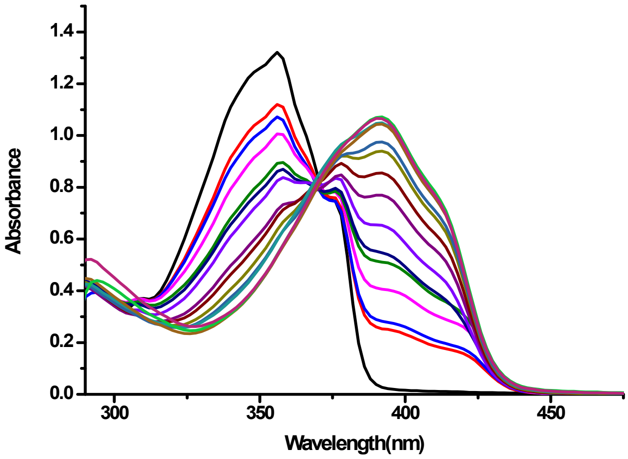

Figure S1. UV-vis absorption spectra of **L** (5.0 x10^{-5} M) in EtOH/H_2O=4:1 solution upon addition Cu^{2+} (0-7.5 x 10^{-5} M) at room temperature.

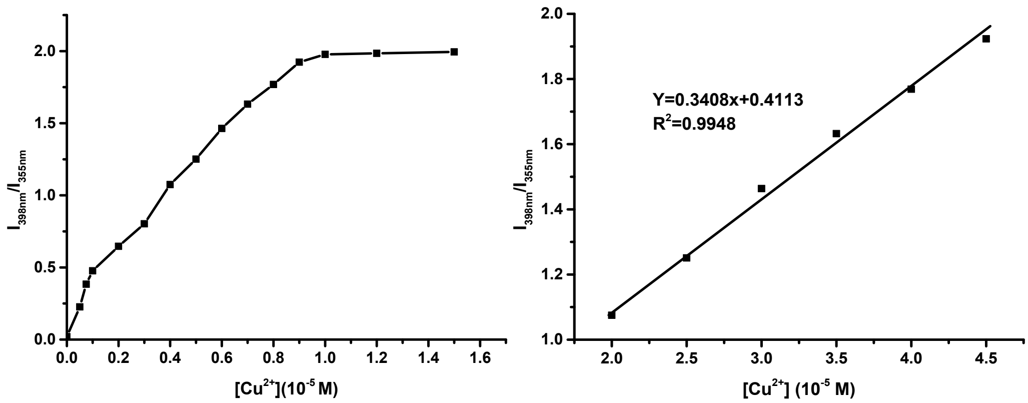

Figure S2. Plot of relative absorbance intensity I_{398nm}/I_{355nm} versus Cu^{2+}(0-7.5x10^{-5} M).

Table S1. Comparison of the recently reported sensors for the determination of Cu^{2+}

Cu^{2+}-sensor	Detection limit (µM)	sensor	Operation mode	Working system	interference	Reference
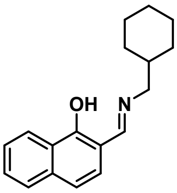	43.11	Fluorescent Sensor	Turn on	CH_3CN	Zn^{2+}	54
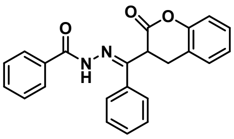	8	Colorimetric /Fluorescent sensor	Turn on	CH_3CN/H_2O (9:1, v/v)	Al^{3+}	55
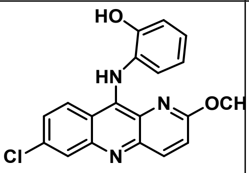	0.16	Colorimetric Sensors	Turn on	CH_3CN/H_2O (V/V, 1: 9)	Fe^{3+}, Cr^{3+}, Al^{3+}	56
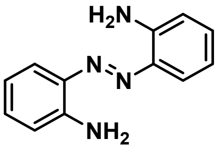	0.648	Colorimetric sensor	Turn on	EtOH	none	57
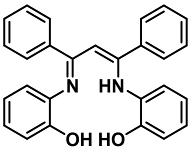	none	Colorimetric Sensors	Turn on	MeOH	Al^{3+}, Mn^{2+}	58
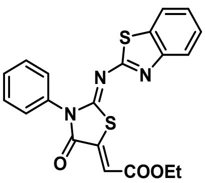	0.36	fluorescent probes	Turn off	CH_3OH/H_2O (50 : 50 v/v)	Hg^{2+}	59
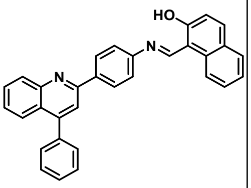	0.044	fluorescent probes	Turn on	in PBS buffer	none	60

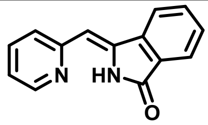	2.82	Colorimetric Sensors	Turn on	EtOH/H_2O	none	This study

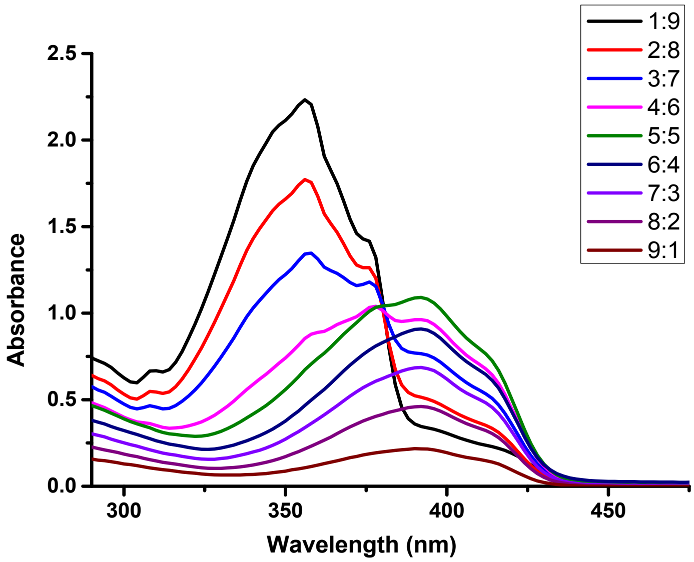

Figure S3. The molar ratio [Cu^{2+}] /[**L**]. The total concentration of Cu^{2+} with receptor L was 5.0×10^{-5} M.

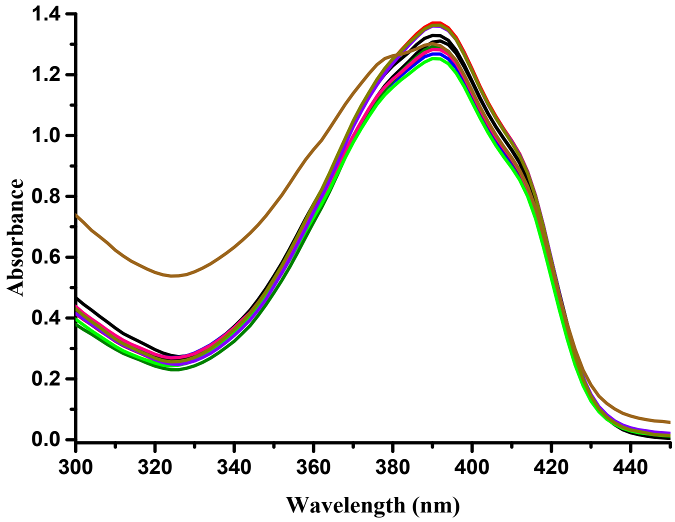

Figure S4. UV–vis selectivity–competition study of receptor **L** with added M(II) salts (5.0 equiv) followed by 1.0 equiv of added Cu(II) ([L] = 5.0×10^{-5} M).

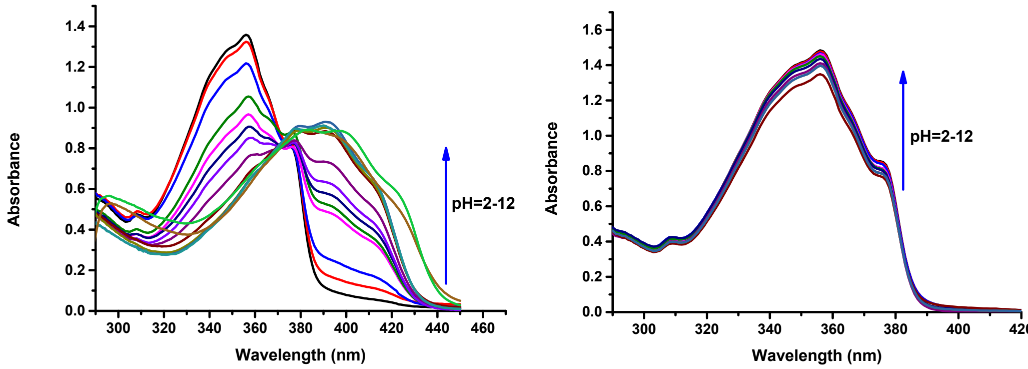

Figure S5. UV-vis absorption spectra of **L**-Cu^{2+}, **L** in EtOH/H_2O=4:1 solution over a range of pH=2-12 at room temperature.

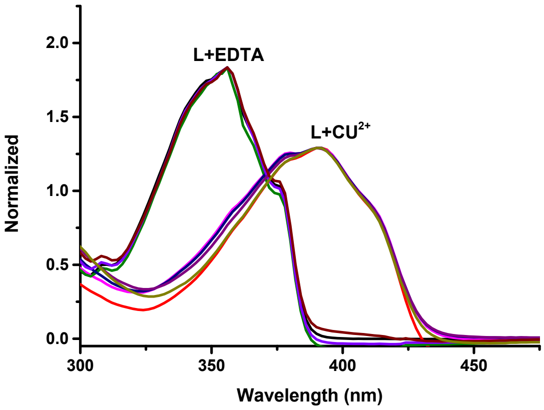

Figure S6. (a) (b)The cycle of **L** -$Cu^{2+}$and Cu^{2+}-EDTA

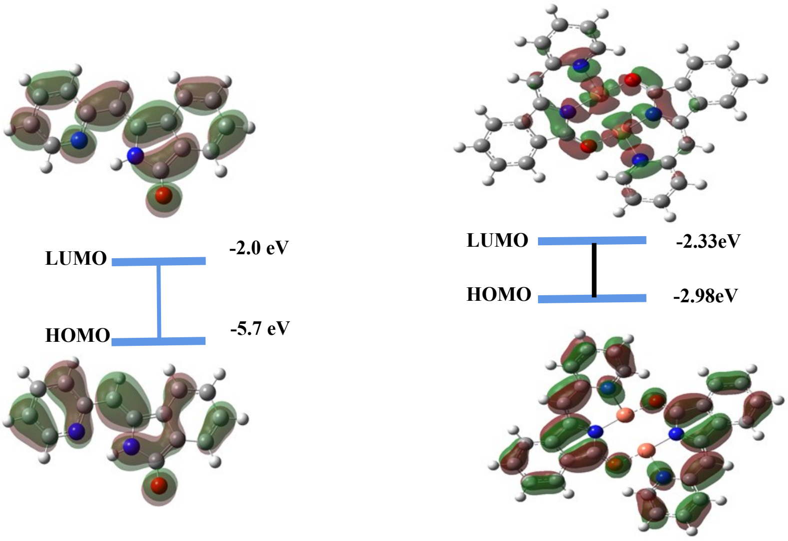

Figure S7. LUMO and HOMO orbitals for **L** and **L**–Cu^{2+}. The red and green lobes represent the

positive and negative signs of the coefficients of the molecular orbitals.

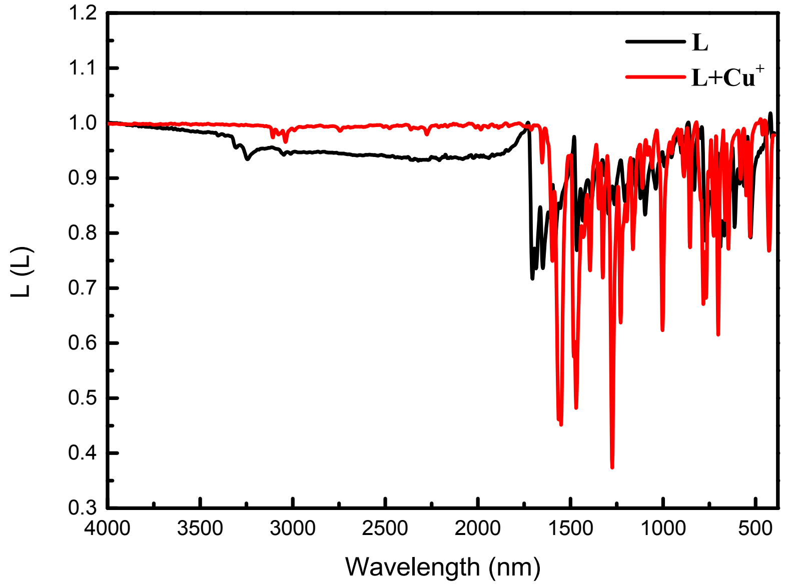

Figure S8. FT-IR spectrum of **L** and [**L**- Cu^{2+}] complex.

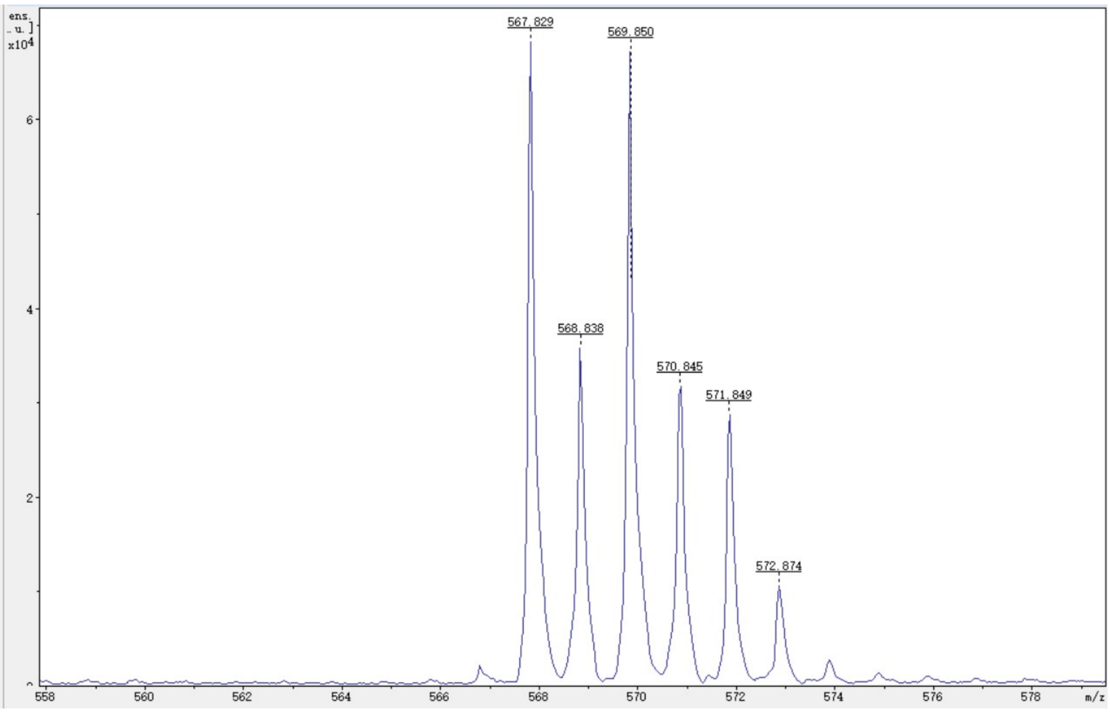

Figure S9. MALDI-TOF spectrum of [**L**+ Cu(II)] complex.
